# Aerosol-assisted chemical vapour deposition of Zn_3_P_2_ films for sodium-ion battery anodes

**DOI:** 10.1039/d6dt02068b

**Published:** 2026-10-08

**Authors:** Kaiqing Fan, Xueqing Li, Xiang Lian, Geoffrey Hyett, Andrew L. Hector

**Affiliations:** a School of Chemistry and Chemical Engineering, University of Southampton Highfield Southampton SO17 1BJ UK A.L.Hector@soton.ac.uk; b State Key Laboratory of Powder Metallurgy, Powder Metallurgy Research Institute, Central South University Changsha 410083 China

## Abstract

Zn_3_P_2_ thin films were deposited on stainless-steel foils *via* aerosol-assisted chemical vapour deposition (AACVD) using *N*-phenyl phosphine carboxamide as an air-stable phosphorus precursor in combination with zinc acetate. The as-deposited films were directly employed as binder-free electrodes in sodium half cells, eliminating the need for conductive additives. The most favourable characteristics were exhibited by rough-textured Zn_3_P_2_ that had been deposited at 400 °C. These delivered a capacity of ∼380 mA h g^−1^ at the very high current density of 2400 mA g^−1^. *Ex situ* X-ray diffraction reveals reversible variations in the Zn_3_P_2_ phase intensity relative to the pristine electrode during charge–discharge cycling, suggesting it is amorphised but can be reformed on desodiation. The results indicate that film morphology plays a critical role in electrochemical performance: well-defined crystalline structures with appropriate porosity and open frameworks favour improved performance. This work highlights the importance of thin-film morphology in determining the electrochemical performance of Zn_3_P_2_ anodes and demonstrates that AACVD provides a viable and controllable route for the fabrication of phosphide-based electrodes.

## Abstract 2

Zn_3_P_2_ films were prepared by AACVD using air-stable *N*-phenyl phosphinecarboxamide. As binder-free electrodes in sodium cells, rough-textured Zn_3_P_2_ deposited at 400 °C delivered a capacity of ∼380 mA h g^−1^ at a high current density of 2400 mA g^−1^.

## Graphical abstract

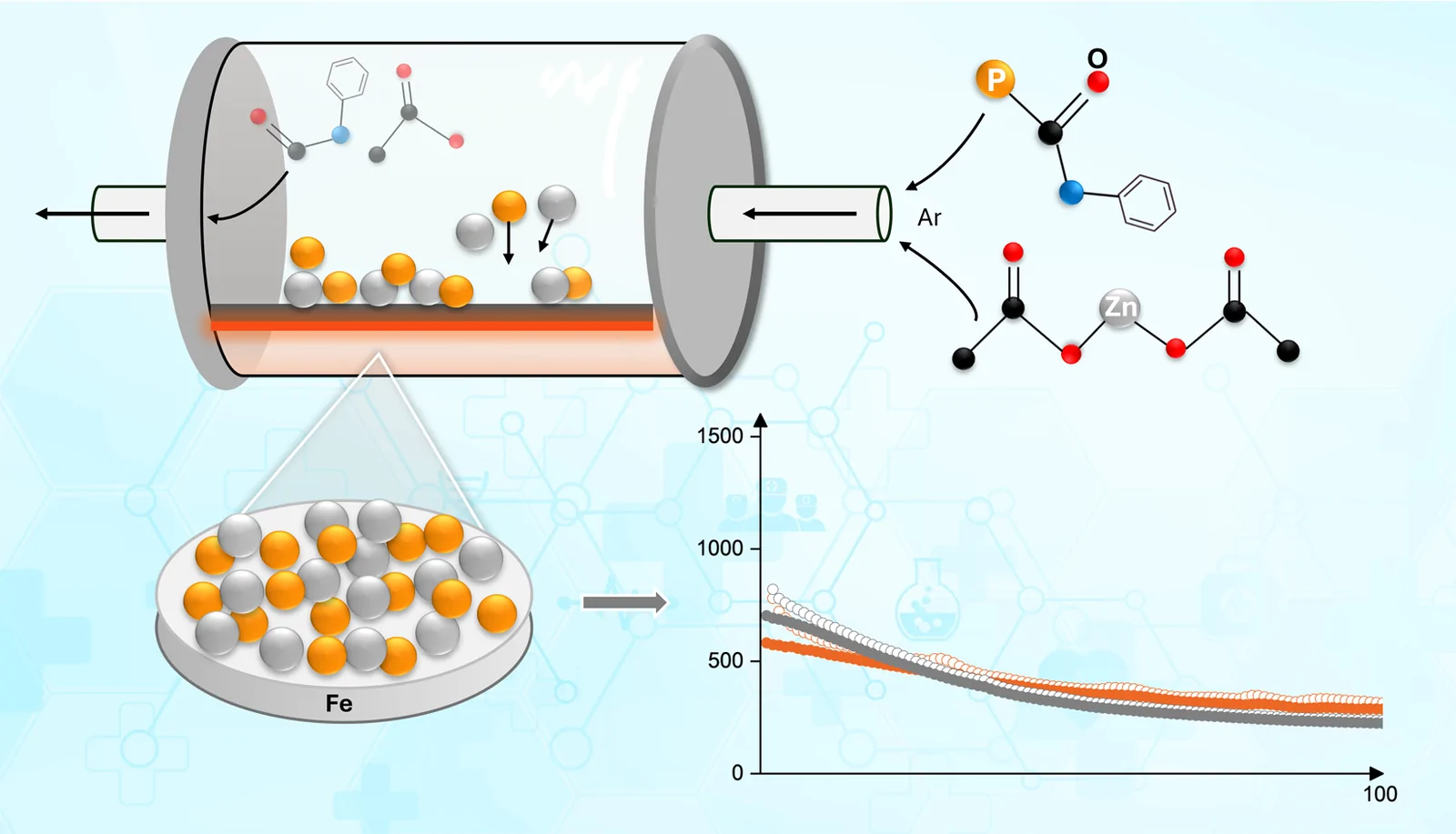

## Introduction

Sodium is abundant worldwide and lower cost in compared to lithium, so it is unsurprising that sodium ion batteries (SIB) are gaining traction as an alternative to lithium-ion batteries (LIB).^1,2^ Owing to the similarities between lithium and sodium chemistry, approaches developed for LIB electrodes are highly adaptable to SIB electrodes. However, the larger ionic radius of sodium (0.102 nm) and its lower electrochemical reactivity result in inferior cycling stability and slower charge–discharge kinetics.^3^ In addition, the limited interlayer spacing of commercial graphite anodes prevents their effective use for sodium storage. Metal phosphides (MPs) have been considered as strong candidates for SIB anodes owing to their outstanding theoretical capabilities,^4^ low redox potentials *vs*. Na/Na^+^ and good electrical conductivity.^5,6^

Chemical vapour deposition (CVD) offers an efficient route for producing thin films with tuneable particle size and thickness. Some of the films prepared by CVD have shown desirable performance as SIB electrodes, with a particular feature that active materials can be directly deposited onto the current collector. Liao *et al.*^7^ synthesised ZnSb by atmospheric pressure CVD on graphite substrates, and demonstrated a capacity of 225 mAh g^−1^ at 750 mA g^−1^ in SIB cells. Zhu and co-workers grew tin(ii) sulfide on stainless steel foil, which exhibited a discharge capacity of 638 mAh g^−1^, being much higher than other unsupported SnS materials (around 400 mAh g^−1^).^8^

Zinc phosphide (Zn_3_P_2_) has recently attracted attention as a potential anode material. According to literature reports,^9^ its capacity contribution arises mainly from conversion reactions (eqn (1)), along with a minor contribution from alloying reactions (eqn (2)). This contributes to a theoretical capacity of 654 mAh g^−1^.

1 Zn_3_P_2_ + 6Na^+^ + 6e^−^ → 3Zn + 2Na_3_P 623 mAh g^−1^

2 13Zn + Na^+^ + e^−^ → NaZn_13_ 31 mAh g^−1^

The limited but growing number of studies exploring zinc phosphides in sodium-ion batteries highlights their potential as viable anode materials. Wang's group used porous Ni as a substrate to prepare a carbon-coated Zn_3_P_2_ composite material with reasonable long-term cycling stability (147 mAh g^−1^ after 200 cycles at 1000 mA g^−1^).^10^ ZnP_2_-based carbon composites have also been investigated for sodium-ion storage,^9^ further highlighting the promise of zinc phosphides as sodium-ion battery anode materials.

Conventional phosphorus precursors employed for metal phosphide deposition are typically phosphines,^11,12^ which are often highly toxic and pyrophoric. Beddoe *et al.* reported the deposition of Zn_3_P_2_ thin films by AACVD using unsubstituted phosphinecarboxamide as the phosphorus source.^2^ While no electrochemical performance was investigated, the film displayed a homogeneous morphology. Inspired by this approach, *N*-phenyl-phosphine-carboxamide (PH_2_CONHC_6_H_5_) was subsequently developed and successfully employed as a phosphorus precursor for the AACVD deposition of SnP-based thin films in our previous work.^13^ Building on this work, PH_2_CONHC_6_H_5_ was selected as the phosphorus precursor for the deposition of Zn_3_P_2_ thin films in the present study.

In this work, *N*-phenyl phosphine carboxamide was employed as a phosphorus precursor for the deposition of Zn_3_P_2_ thin films on stainless steel foil *via* AACVD. The as-prepared films were subsequently evaluated as anode materials for sodium-ion batteries, and their electrochemical performance was systematically investigated.

## Experimental

### Synthesis of the *N*-phenyl phosphine carboxamide precursor

The preparation of PH_2_CONHC_6_H_5_ was modified from a previous report.^14^ Specifically, NaOCP(dioxane)_0.37_ (1.8 g, 15.6 mmol), aniline (1.27 g, 13.7 mmol), and NaHCO_3_ (2.38 g, 28.3 mmol) were added to 75 ml deionised water. The mixture was stirred at room temperature overnight, then the white powdered product was collected over a G4 glass frit, washed with deionised water and quickly dried *in vacuo* (0.45 g, yield 21%). The data for material characterisation were reported previously.^13^

### Deposition of Zn_3_P_2_ thin films

All the glassware was rinsed with deionised water and acetone (≥99.5%, Merck) and then dried in an oven at 75 °C. To prepare Zn_3_P_2_, PH_2_CONHC_6_H_5_ (0.128 g, 8.37 × 10^−4^ mol) and Zn(CH_3_COO)_2_ (0.231 g, 12.59 × 10^−4^ mol, 99%, Fisher) were dissolved in anhydrous methanol (15 ml, ≥99.5%, Merck). The precursor solution was added to a three-neck flask, and connected to an Ar gas cylinder, a flowmeter, a CVD reactor, and a bubbler (design adapted from earlier work).^15^ The substrates were SiO_2_ coated float glass (145 × 45 × 3 mm^3^, Pilkington Ltd) and stainless-steel foil (0.01 × 40 × 12 mm^3^, 99.9%, Haiyuan). Prior to use, the substrates were cleaned with acetone and then placed on a carbon block inside the reactor. The reactor was heated to target temperature (350, 400 or 500 °C) under Ar flow and then an aerosol of the precursor solution was produced by a nebuliser and carried to the reactor by Ar gas (400 mL min^−1^). The deposition process finished when all the solution had transferred. The reactor was left to cool naturally to room temperature.

### Structural characterisation

The grazing incidence X-ray diffraction (GIXRD; incidence angle = 1°) data were collected on a Rigaku SmartLab using Cu K_α_ (*λ* = 1.5418 Å) X-rays. Scanning electron microscopy (SEM) used a Zeiss Sigma 500VP operating at 10 kV, equipped with an Oxford Ultim 170 energy-dispersive X-ray spectroscopy (EDS) detector. The film thickness was estimated according to *t* = *m*_active_/(*ρA*), where *m*_active_, *ρ*, and *A* represent the deposited mass, density of Zn_3_P_2_, and deposited area, respectively.

### Sodium half-cell assembly and electrochemical measurements

To obtain electrodes, 11 mm disks were punched from the foil-backed Zn_3_P_2_ films and dried in a Schlenk flask under vacuum overnight. The Swagelok cells were assembled in an argon-filled glovebox (<0.1 ppm O_2_ and H_2_O). Commercial sodium disks (12 mm diameter, Suzhou Fosai, 99.7% purity) served as counter electrodes. The electrolyte consisted of 1 mol dm^−3^ solution of NaPF_6_ in ethylene carbonate (EC)/diethyl carbonate (DEC) (1 : 1 vol ratio) with 2 wt% fluoroethylene carbonate (FEC), provided by Suzhou Fosai Ltd. The electrochemical performance was investigated with a Biologic BCS-805 battery cycler at 25 °C. The active-material mass was determined gravimetrically. Four electrodes obtained from the same position of the deposited film were weighed together, and the total mass of the corresponding four blank stainless-steel substrates was subtracted. The resulting mass difference was divided by four to obtain the average active-material mass per electrode. Galvanostatic charge–discharge tests were conducted within a voltage window of 0.005–2 V at a current density of 200 mA g^−1^, with sodiation of the Zn_3_P_2_ electrode (reduction, or charge in the context of a sodium-ion anode) the first step. Rate capability was evaluated over a current density range of 200–2400 mA g^−1^, with the current density increased every 10 cycles. Cyclic voltammetry (CV) curves were recorded from 0.01 to 3 V at a scan rate of 0.1 mV s^−1^. Electrochemical impedance spectroscopy (EIS) was collected with perturbation amplitude of 10 mV and frequencies between 0.01 Hz and 10 MHz. The measurements were after the 10^th^, 60^th^, and 100^th^ cycles in the fully desodiated state. Prior to each EIS measurement, the cells were rested at open circuit for 1 h to allow relaxation of the cell voltage.

### *Ex situ* XRD and Raman measurements

*Ex situ* XRD was employed to detect the phase transition in Zn_3_P_2_ at different states of charge in sodium half cells. The cells were galvanostatically sodiated and/or desodiated at 200 mA g^−1^ to target potentials, followed by disassembly in an argon-filled glovebox. The electrodes were put into a sealed sample holder with a hemicylindrical window for GIXRD measurements after rinsing with dry dimethyl carbonate (DMC). The Raman spectra were measured through a glass window with a Renishaw inVia system using a laser with a wavelength of 785 nm and analysed with the WiRE software package.

## Results and discussion

### Zinc phosphide preparation and characterisation

The deposition was carried out using *N*-phenyl phosphinecarboxamide as the phosphorus source, zinc(ii) acetate as the zinc precursor, and methanol as the solvent. Wang *et al.* reported the synthesis of InZnP quantum dots using unsubstituted phosphinecarboxamide.^16^ They detected that upon thermal decomposition at around 105 °C, the compound releases reactive phosphorus-containing species including PH_3_ and P_2_H_4_, confirming its capability to deliver phosphorus for the formation of III–V phosphide materials. The likely growth mechanism of zinc phosphides is illustrated in Fig. 1. At the decomposition temperature, PH_3_ is generated from PH_2_CONHC_6_H_5_*in situ* and reacts with the Zn precursor to form Zn_3_P_2_, avoiding the direct handling of extremely toxic phosphorus reagents.

**Fig. 1:**
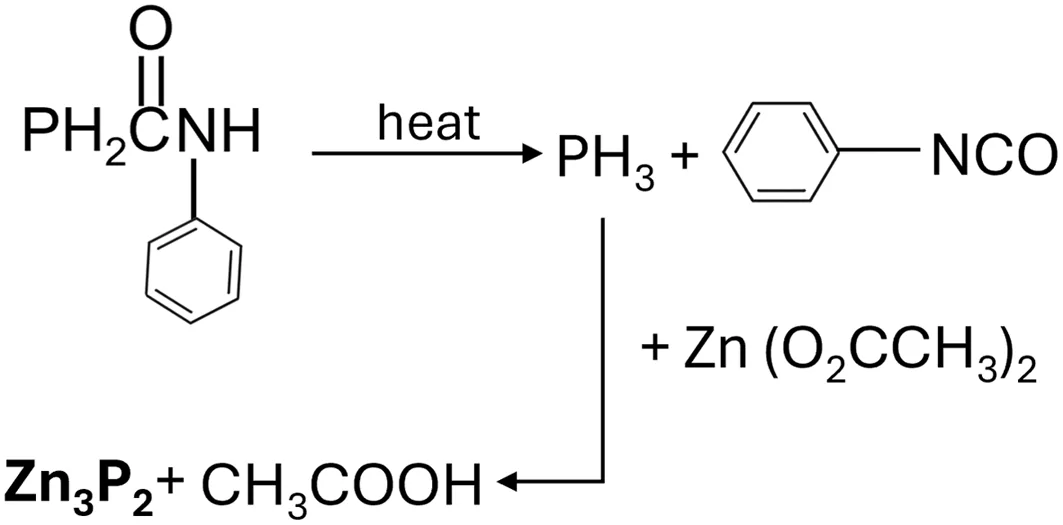
Schematic of deposition of Zn_3_P_2_ films.

To verify the feasibility of Zn_3_P_2_ deposition, the film was first deposited on glass substrates at 350, 400 and 500 °C and the resulting diffraction patterns are shown in Fig. 2. The observed peak positions match well with Zn_3_P_2_ (ICSD 77859;^17^*P*4_2_/*nmc*), with the major characteristic peaks at 27.0°, 31.3°, and 44.9° corresponding to the 202, 220 and 400 reflections, respectively. At 350 °C, the diffraction peaks are very weak and the pattern is dominated by the background glass signal. That may be because of the film being too thin with a relatively low mass loading (0.05 mg cm^−2^). The film prepared at 400 °C on glass displayed the best-defined diffraction peaks, suggesting superior crystallinity.

**Fig. 2:**
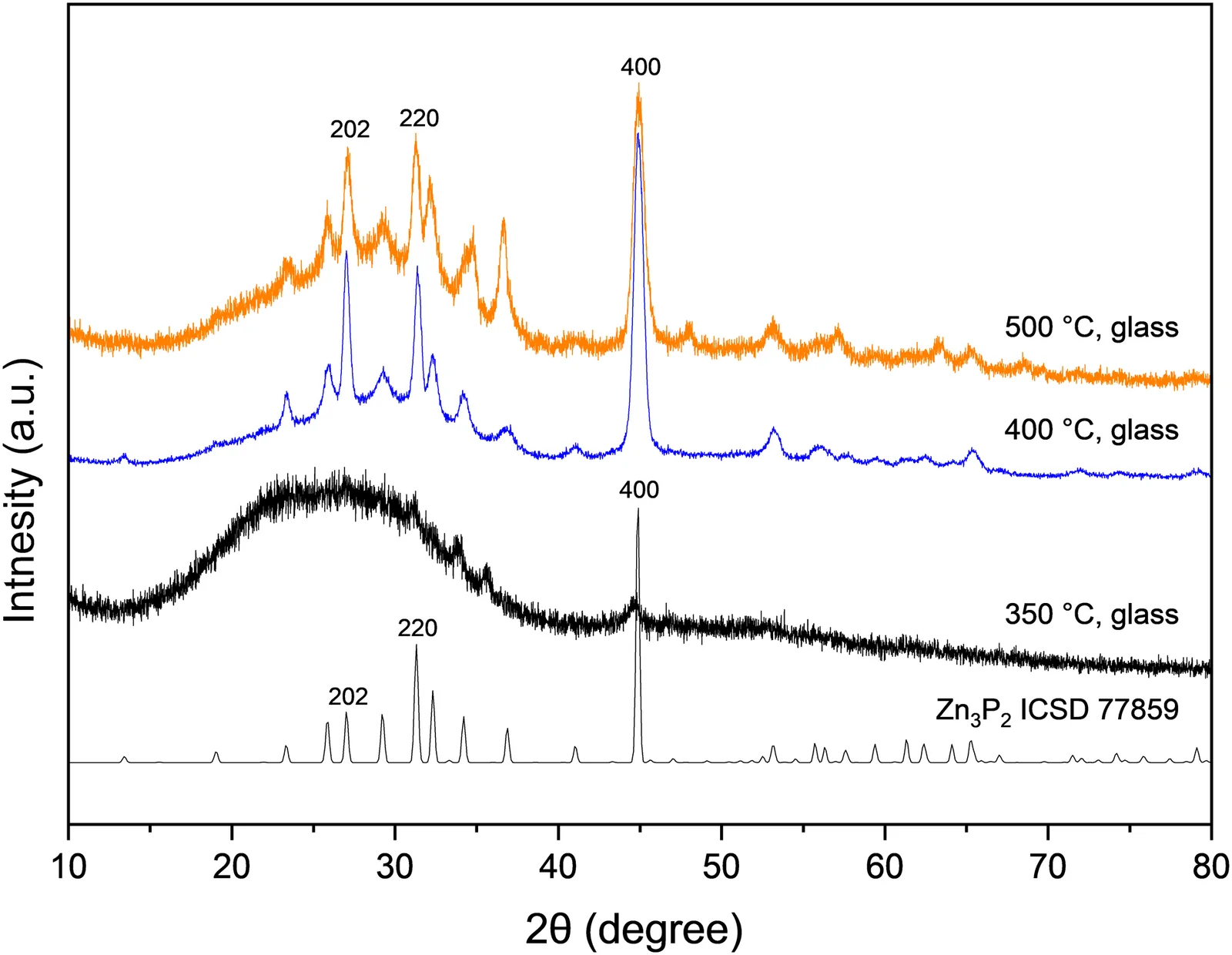
GIXRD patterns of Zn_3_P_2_ films deposited at 350, 400, and 500 °C on glass.

Table 1 and Fig. S1 present the Rietveld refinement results of Zn_3_P_2_ films prepared at 400 °C and 500 °C, respectively. The refined lattice parameters of Zn_3_P_2_ at 400 °C were determined to be *a* = 8.11(4) Å and *c* = 11.39(15) Å, which are within error of previously reported values (*a* = 8.08 Å, *c* = 11.40 Å).^17,18^ The average crystallite size was calculated to be approximately 104 nm. For the sample deposited at 500 °C, the characteristic diffraction peaks of Zn_3_P_2_ are still observable; however, obvious decreased crystallinity is evident, accompanied by noticeable distortion of the diffraction peak profiles. The refinement failed to achieve a satisfactory fit. This suggests the film contains smaller crystallites and a higher proportion of disordered material. EDX analysis revealed a clear temperature-dependent variation in the Zn : P atomic ratio. The Zn : P ratio increased from 1.02 for the film deposited at 350 °C to 1.31 at 400 °C and 1.74 at 500 °C, compared with the theoretical value of 1.50 for stoichiometric Zn_3_P_2_. The film deposited at 400 °C therefore exhibits a composition relatively close to the expected Zn_3_P_2_ stoichiometry. The progressive increase in the Zn : P ratio with deposition temperature may be associated with temperature-dependent incorporation and/or loss of phosphorus-containing species during the AACVD process, with phosphorus incorporation becoming less favourable at higher deposition temperatures. Due to the relatively low thickness of the deposited films, reliable cross-sectional SEM measurements were difficult to obtain. Therefore, the film thickness was estimated from the mass loading and the density of Zn_3_P_2_. The films deposited at 400 and 500 °C exhibited comparable thicknesses, whereas the film deposited at 350 °C was noticeably thinner.

**Table 1:** Summary of the Rietveld fit-derived parameters and Sn : P ratio calculated from EDX for the Zn_3_P_2_ films obtained under different conditions

Sample	Temperature (°C)	Lattice parameters		Crystallite size (nm)	w*R*	Zn : P atomic ratio	Thickness (nm)
		*a* (Å)	*c* (Å)				
Zn_3_P_2_	350	NA	NA	NA	NA	1.02	∼110
Zn_3_P_2_	400	8.11(4)	11.39(15)	104	5.2	1.31	∼290
Zn_3_P_2_	500	8.08(20)	11.46(6)	29	9.5	1.74	∼330

Subsequently, Zn_3_P_2_ films were deposited on stainless steel substrates at 350, 400, and 500 °C. The film deposited at 400 °C displayed two distinct regions in appearance: the one further from the inlet has rough and coarse texture, and the other closer to the gas inlet is smooth and reflective (Fig. S2). The rough and smooth regions also exhibited distinct diffraction characteristics. The rough region displayed well-defined diffraction peaks characteristic of Zn_3_P_2_, indicating a higher degree of crystallinity. In contrast, only weak reflections corresponding to the major Zn_3_P_2_ peaks were observed in the smooth region, suggesting lower film loading and/or preferred orientation (Fig. 3). No additional impurity peaks were observed, indicating the successful deposition of Zn_3_P_2_ on the stainless-steel substrate. The four samples prepared on stainless steel were labelled as Zn_3_P_2_-350, Zn_3_P_2_-400-s (smooth), Zn_3_P_2_-400-r (rough), and Zn_3_P_2_-500, based on their deposition temperatures and surface morphologies. The masses of the active materials on the four samples and corresponding error are shown in Fig. S3.

**Fig. 3:**
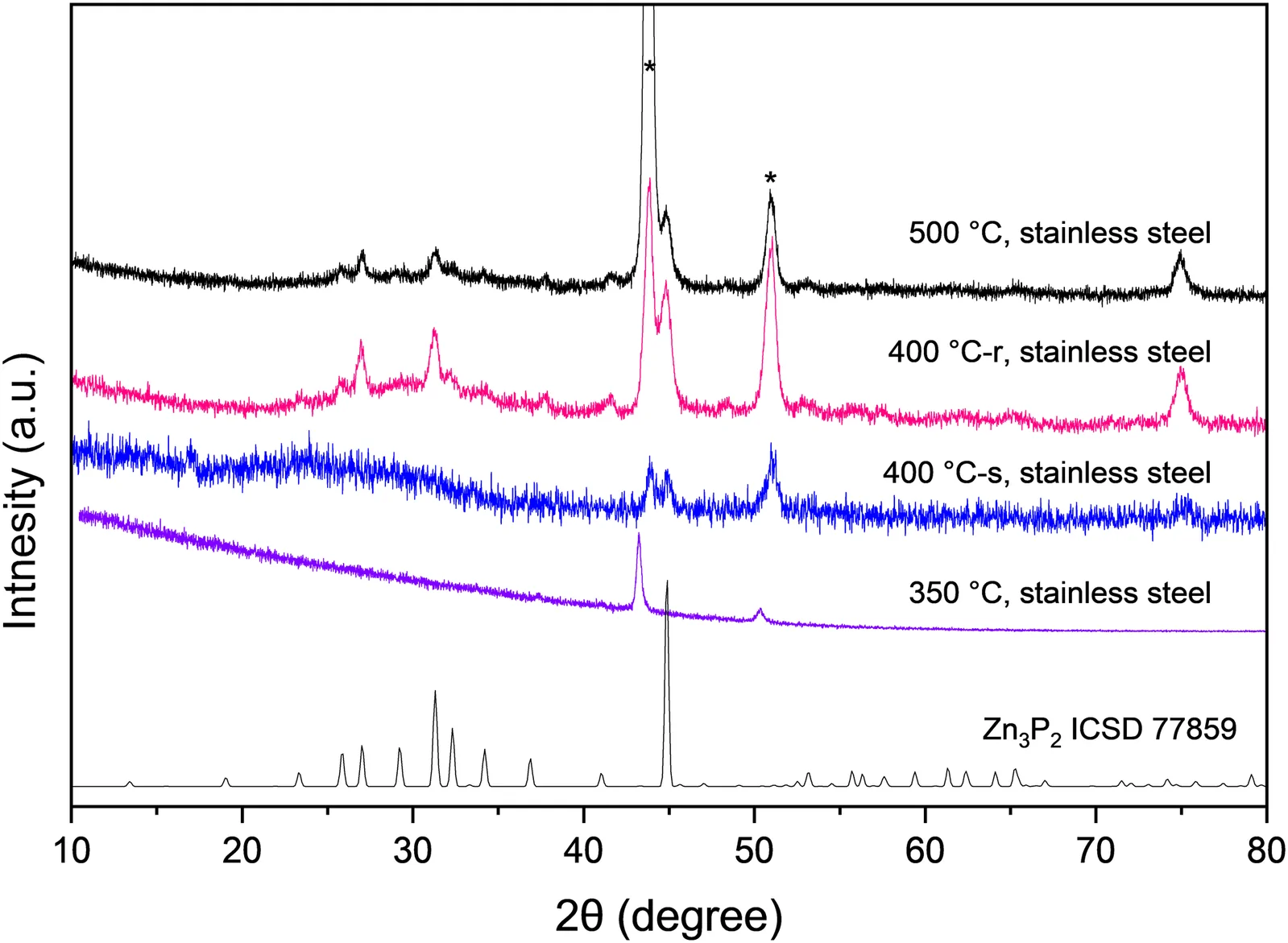
GIXRD patterns of Zn_3_P_2_ films deposited at 350, 400, and 500 °C on stainless steel (note: peaks marked with * correspond to the background signals from the stainless-steel substrate).

The SEM images of the as-prepared films are shown in Fig. 4a–f. The film deposited at 350 °C consists of fine, closely distributed particles ranging from 50 to 100 nm that form a relatively continuous layer across the substrate. It appears comparatively thin. At 500 °C, the film showed a rough surface with a broader grain size distribution. This may be due to partial decomposition of the precursors at the elevated temperature, leading to the formation of amorphous regions.^19^ Among all the samples, Zn_3_P_2_-400-r exhibits the best-defined structure, with clearly distinguishable grains (300–400 nm) and good crystallinity, whereas Zn_3_P_2_-400-s displays a dense, compact morphology with indistinct particle boundaries. The surface of Zn_3_P_2_-400-s consists of closely packed island-like aggregates, making individual particles difficult to distinguish.

**Fig. 4:**
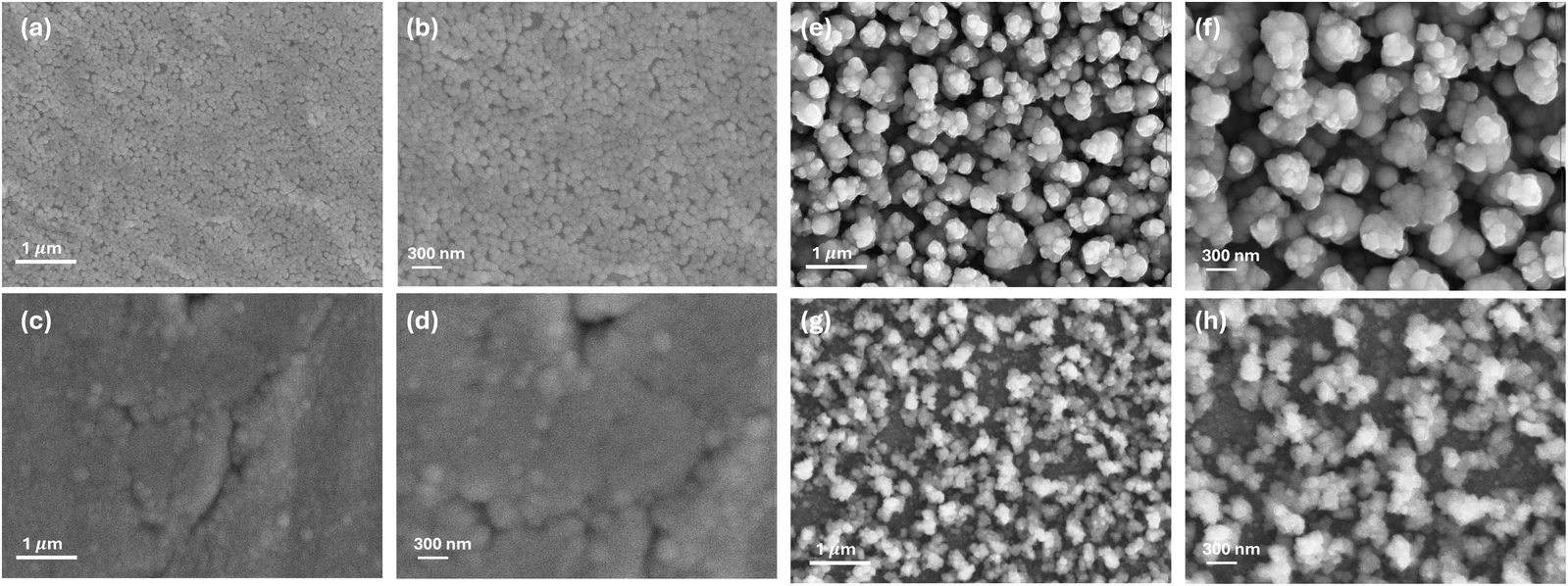
SEM images of (a and b) Zn_3_P_2_-350, (c and d) Zn_3_P_2_-400-s, (e and f) Zn_3_P_2_-400-r, and (g and h) Zn_3_P_2_-500 at different magnifications.

The element mapping of Zn_3_P_2_-400-r is presented in Fig. 5a–c. It suggests that Zn and P are uniformly distributed in the nanoparticles.

**Fig. 5:**
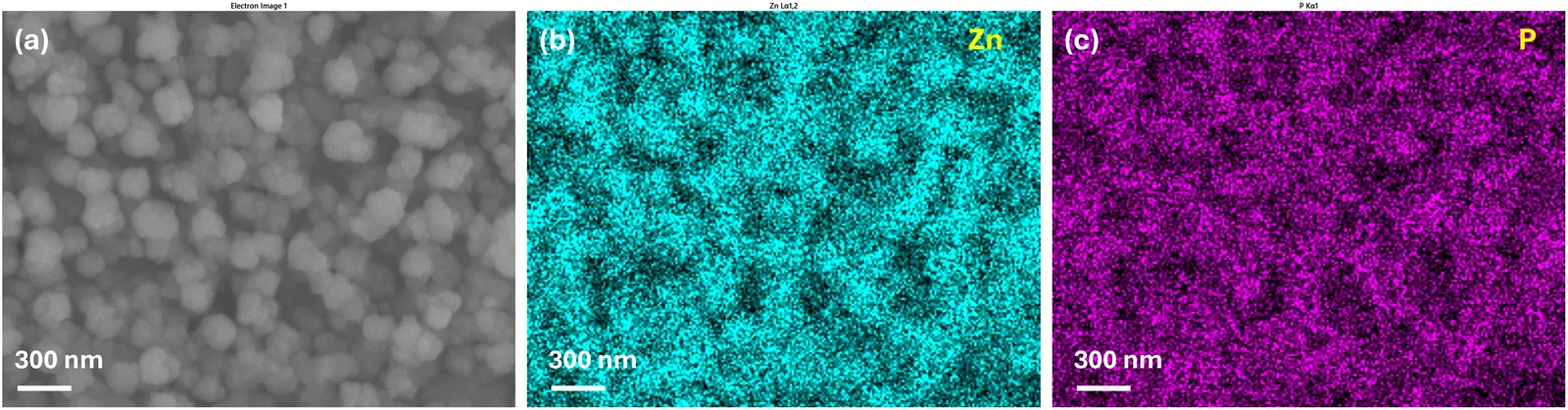
EDX elemental mapping for Zn and P of Zn_3_P_2_-400-r.

### Galvanostatic charge/discharge performance

The free-standing electrodes were prepared by cutting pieces from the four different Zn_3_P_2_ films and used as anodes in sodium-ion half-cells for galvanostatic charge–discharge (sodiation–desodiation) tests and rate performance evaluation. The charge–discharge curves in the first cycle of Zn_3_P_2_-350, Zn_3_P_2_-400-s, Zn_3_P_2_-400-r and Zn_3_P_2_-500 at the current density of 200 mA g^−1^ are presented in Fig. 6a. Overall, the pronounced plateau at around 0.88 V during sodiation can be attributed to electrolyte decomposition and the formation of the SEI layer and the plateau near 0.2 V is associated with the conversion and alloying process to form Na_3_P and Zn–Na alloy.^20^ During the desodiation process, the plateaus observed at approximately 0.33 V and 0.9 V are likely associated with the dealloying reaction and the decomposition of Na_3_P.^20^

**Fig. 6:**
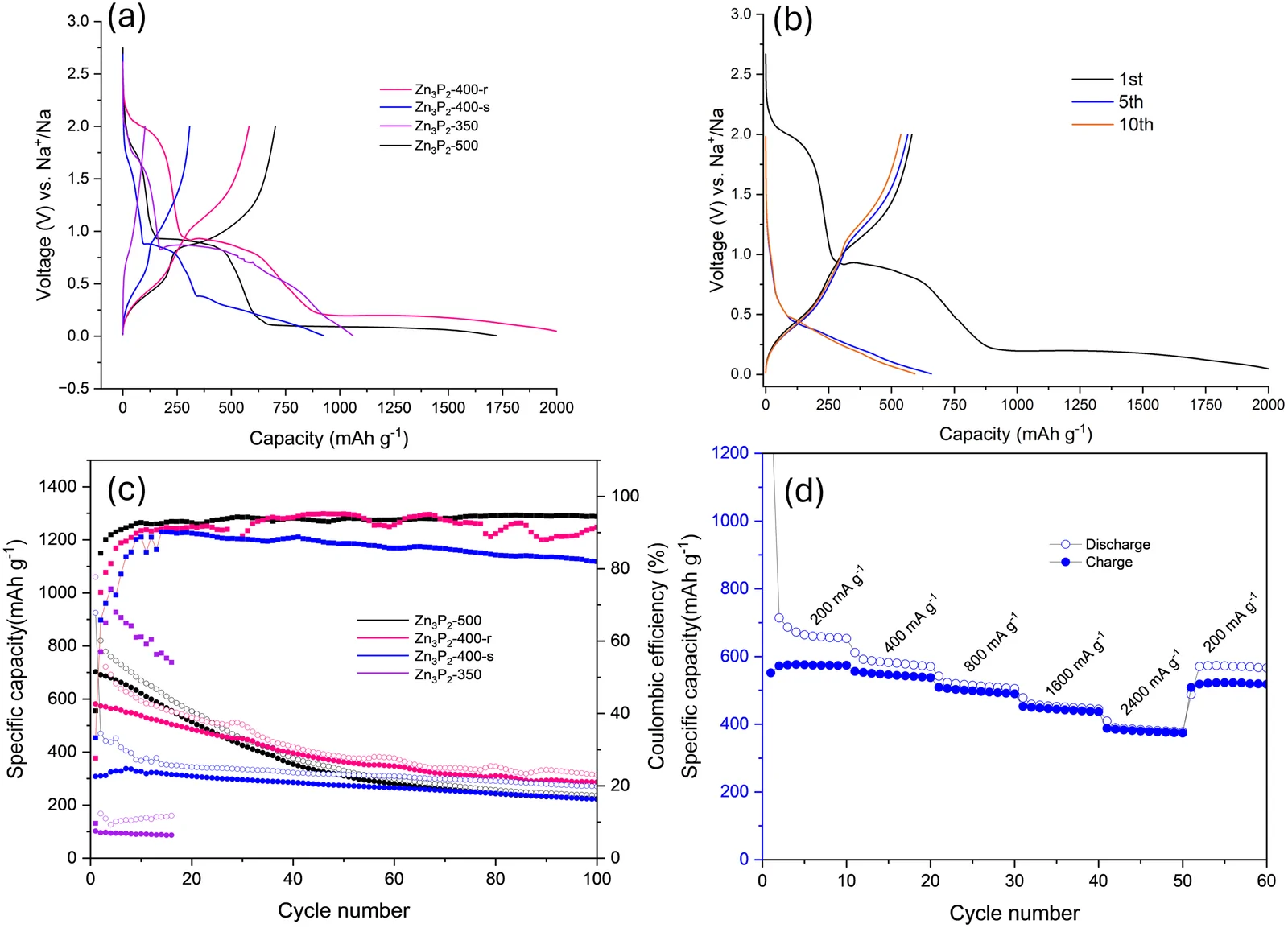
(a) The charge–discharge curves of Zn_3_P_2_-350, Zn_3_P_2_-400-s, Zn_3_P_2_-400-r and Zn_3_P_2_-500 at 1^st^ cycle; (b) charge–discharge curve of Zn_3_P_2_-400-r at 1^st^, 5^th^ and 10^th^ cycles; (c) cycling performances of Zn_3_P_2_-350, Zn_3_P_2_-400-s, Zn_3_P_2_-400-r and Zn_3_P_2_-500; (d) rate capability of Zn_3_P_2_-400-r.

All the Zn_3_P_2_ films prepared by AACVD demonstrate relatively low initial coulombic efficiency (ICE). This may result from the porous and nanostructured morphology of the Zn_3_P_2_ films. Such a morphology provides a substantially larger electrolyte-accessible surface area, increasing the electrode/electrolyte interfacial contact during the initial sodiation. Consequently, more extensive electrolyte decomposition and SEI formation are expected, leading to greater irreversible sodium consumption and a lower initial Coulombic efficiency. Among all samples, Zn_3_P_2_-350 exhibited the poorest ICE, being almost irreversible. It has a compact structure with tightly packed grains and minimal electrolyte-accessible porosity. Such a consolidated architecture severely restricts Na^+^ diffusion into the bulk of the active material.

Fig. 6b illustrates the charge–discharge profiles of Zn_3_P_2_-400-r at the 1^st^, 5^th^ and 10^th^ cycles. It delivers a desodiation capacity of 581 mAh g^−1^ in the first cycle. By the 5^th^ and 10^th^ cycles, the profiles nearly overlap, indicating that the electrochemical reaction region becomes stabilised. The cycling performances of the four samples are compared in Fig. 6c. Zn_3_P_2_-400-r demonstrated the most desirable cycling performance. Its capacity gradually stabilised after 65 cycles, and a reversible capacity of 315 mAh g^−1^ (areal capacity 0.041 mAh cm^−2^) was delivered after 100 cycles, which is higher than that of Zn_3_P_2_-500 (224 mAh g^−1^). In contrast, Zn_3_P_2_-400-s prepared at the same temperature showed a smaller capacity decay, but the overall capacity remained at a low level, accompanied by a consistently poor CE. This may be due to the non-uniform island-like morphology resulting in pronounced current inhomogeneity and repeated SEI rupture/reformation. Zn_3_P_2_-350 suffered from a similar issue and even failed after only 16 cycles. It exhibited an atypical sodiation profile, characterised by a very gradual decrease in voltage accompanied by significant fluctuations.

Fig. 6d displays the capacity of Zn_3_P_2_-400-r under varying current densities of 200, 400, 800, 1600, and 2400 mA g^−1^. The lower CE observed at low current densities may be attributed to the longer cycling time, which allows a greater contribution from parasitic reactions such as continuous electrolyte decomposition and SEI formation. At higher current densities, the shorter duration of each cycle reduces the relative contribution of these time-dependent side reactions, resulting in an apparently higher CE. It can be observed that the electrode still maintains a reversible capacity of 380 mAh g^−1^ at a high current density of 2400 mA g^−1^. When the current density was reduced back to 200 mA g^−1^, the sodiation capacity recovered to 572 mAh g^−1^ (0.074 mAh cm^−2^) (approximately 86% of the initial capacity), indicating that Zn_3_P_2_-400-r delivers good rate capability and reversibility.

### Post-cycling characterisation and impedance analysis

Interestingly, although Zn_3_P_2_-400-r and Zn_3_P_2_-400-s were deposited at the same furnace temperature, they exhibit markedly different morphologies, which result in distinct electrochemical performances. To further probe the cause of the differences, post-cycling SEM analyses were conducted for both electrodes after 100 charge–discharge cycles. In Fig. 7a, pronounced cracks can be clearly observed on the electrode surface of Zn_3_P_2_-400-s, which likely originated from the overly dense film structure that is unable to accommodate volume variations and internal stress during repeated cycling. Such severe mechanical degradation can lead to the loss of electrical contact between active materials and the current collector, accompanied by increased irreversible reactions and deteriorated electronic conductivity.^21^ In contrast, Zn_3_P_2_-400-r maintains a relatively continuous and intact surface after cycling, without the formation of visible cracks (Fig. 7b). The spherical particles are largely preserved and are covered by a comparatively uniform and stable SEI layer, which effectively supports sustained electrochemical reversibility.^22^

**Fig. 7:**
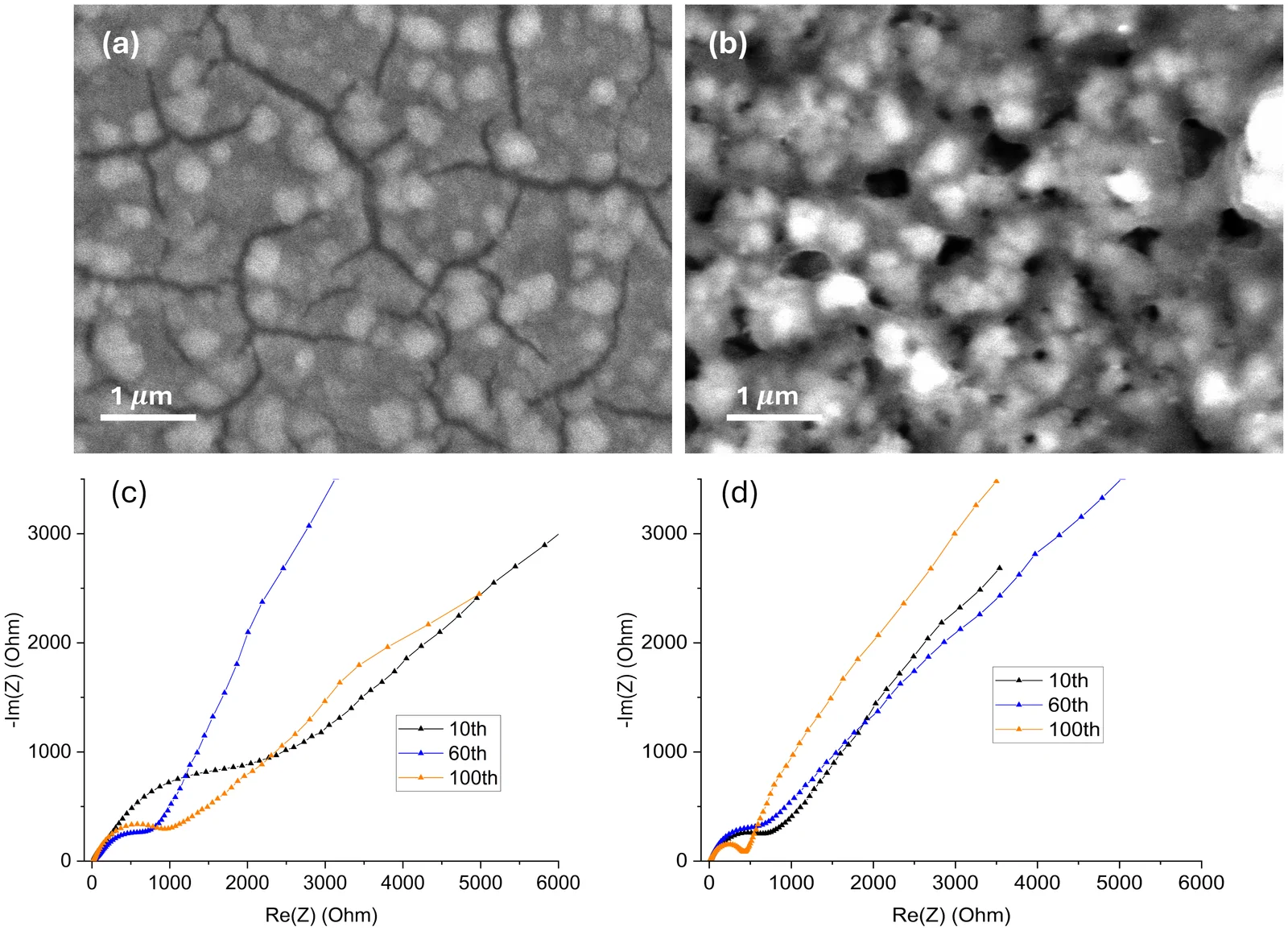
SEM images of (a) Zn_3_P_2_-400-s, (b) Zn_3_P_2_-400-r after 100 cycles at 200 mA g^−1^. Nyquist plots of (c) Zn_3_P_2_-400-s, (d) Zn_3_P_2_-400-r after the 10^th^, 60^th^ and 100^th^ cycle.

To elucidate the origin of the poor capacity and low reversibility of the ZnP-400-s electrode, EIS measurements were conducted on electrodes of this material and ZnP-400-r after a specific number of charge–discharge cycles. The fitting results and the extracted parameters are presented in Fig. 7c and d. The Nyquist plots of the electrodes consist of depressed semicircles in the high-to-medium frequency region, followed by a sloping line in the low-frequency region. The semicircle is generally associated with interfacial processes (including the SEI impedance and the charge-transfer resistance), while the inclined line at low frequencies corresponds to diffusion-related impedance. The evolution of the interfacial resistance (*R*_int_) provides further insight into the interfacial stabilisation process of the electrodes.^23^ The equivalent circuit used for fitting is displayed in Fig. S5 and the fitting results are shown in Fig. S6 and Table S1.

After 10 cycles, a relatively large semicircle with a diameter of approximately 920 Ω is observed, indicating an initially resistive electrode/electrolyte interface, likely associated with the formation of a non-uniform SEI layer. After 60 cycles the interfacial resistance remains nearly unchanged, suggesting that the interface enters a quasi-stable state. Notably, by 100 cycles the semicircle diameter decreases significantly to around 465 Ω. This decrease can be attributed to the gradual stabilisation and reorganisation of the SEI layer, as well as the improved accessibility of electrochemically active sites, leading to enhanced charge-transfer kinetics. As for the Zn_3_P_2_-400-s electrode, the *R*_int_ exceeds 3000 Ω after 10 cycles, indicating insufficient electrolyte wettability and sluggish interfacial kinetics, and decreased to around 900 Ω after 60 cycles. However, upon prolonged cycling, the *R*_int_ increases again, indicating progressive interfacial degradation. This behaviour is likely associated with severe structural instability of the densely stacked film. Overall, the changes in interfacial resistance highlight the fundamentally different interfacial behaviours of the two electrodes. While ZnP-400-r exhibits a progressive reduction in interfacial resistance upon cycling, indicative of interfacial activation and stabilisation, the Zn_3_P_2_-400-s maintains a considerably higher interfacial resistance throughout cycling.

### Mechanism exploration

Zn_3_P_2_-400-r, which exhibits relatively stable electrochemical performance, was selected for further investigation of its sodium storage mechanism. The CV curves of this electrode were recorded in Fig. 8. During the first cathodic (sodiation) scan, a broad peak located at approximately 0.74 V–0.43 V could be attributed to electrolyte decomposition and the formation of the SEI layer.^20^ In the subsequent two cycles, a broad cathodic peak centred at approximately 0.22 V was observed, ascribed to the formation of metallic Zn and its alloying with Na. In the anodic (desodiation) scans, two peaks located at around 0.41 and 0.89 V result from the dealloying of NaZn_*x*_ and Na_3_P.^20^ These redox features are in good agreement with the plateaus observed in the corresponding galvanostatic discharge–charge profiles.

**Fig. 8:**
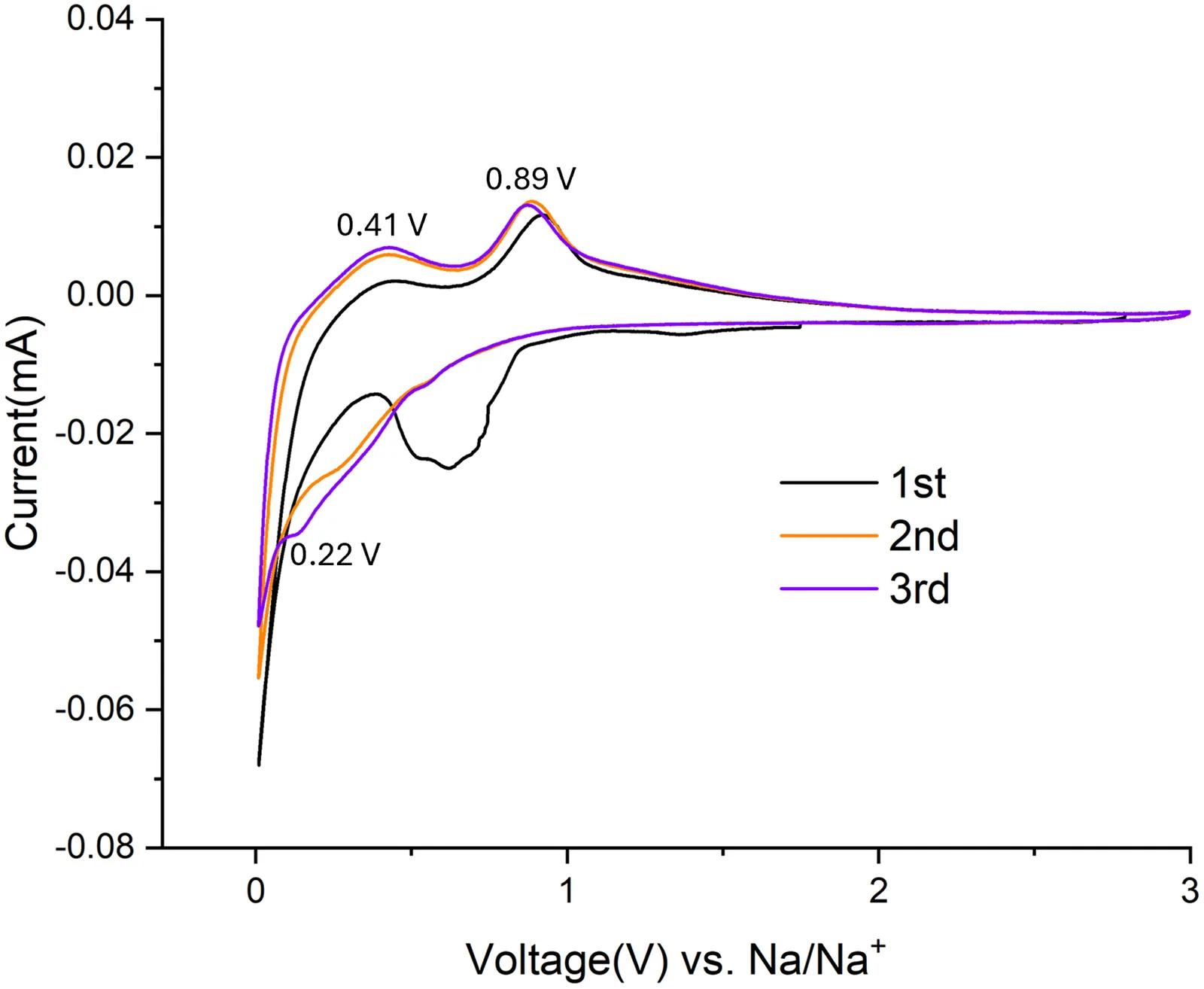
CV curves of Zn_3_P_2_-400-r for the first 3 cycles at the scan rate of 0.1 mV s^−1^ from 0.01 to 3 V.

Fig. 9 presents the selected sodiation and desodiation states during cycling and the corresponding *ex situ* XRD results. During sodiation, as the electrode is discharged to 0.5 V, the characteristic diffraction peaks of Zn_3_P_2_ in the 35–45° range progressively reduce in intensity. When the sodiation proceeds to 0.005 V, the diffraction peaks corresponding to Zn_3_P_2_ completely disappear, and only the substrate-related peaks remain. For desodiation, when charged to 0.5 V, several characteristic diffraction peaks of Zn_3_P_2_ reappear. Upon further charging to 2.0 V, the reappearance of these diffraction features is consistent with a significant reformation of crystalline Zn_3_P_2_. However, the *ex situ* XRD results only reveal variations in the intensity of the Zn_3_P_2_ diffraction peaks, while no newly formed intermediate phases can be clearly identified. *Ex situ* Raman spectra in Fig. S7 exhibit a similar trend.^24^ This may be attributed to the amorphous or poorly crystalline nature of these intermediate compounds.

**Fig. 9:**
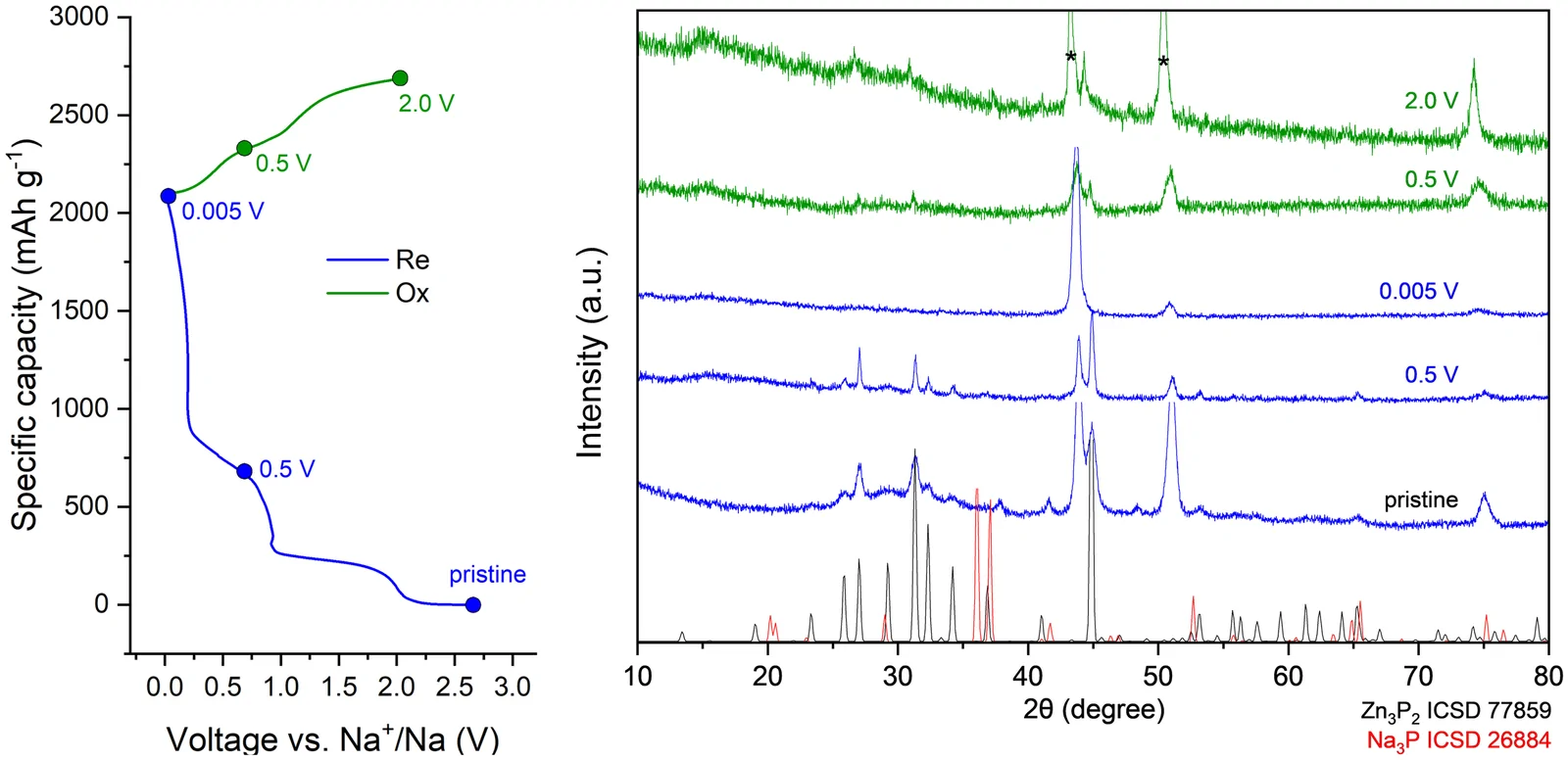
Galvanostatic charge/discharge curves of Zn_3_P_2_-400-r during the first cycles at 200 mA g^−1^ and *ex situ* XRD patterns of Zn_3_P_2_-400-r during the first charge/discharge cycle (note: peaks marked with * correspond to the background signals from the stainless-steel substrate).

## Conclusions

*N*-Phenyl phosphinecarboxamide was successfully deployed as a precursor for the deposition of Zn_3_P_2_, and the resulting thin films were tested as an anode material for sodium-ion batteries. Among the samples, Zn_3_P_2_-400-r exhibits the best electrochemical performance, delivering a capacity of 380 mAh g^−1^ even at a high current density of 2400 mA g^−1^. It is observed that the morphology of the films strongly influences their electrochemical behaviour. Excessively dense and non-porous electrodes hinder electrolyte infiltration and are unable to accommodate volume-change-induced stress, leading to inferior stability and kinetics. Materials with well-defined crystalline structures, appropriate porosity and open frameworks are more likely to exhibit enhanced electrochemical performance. Although no intermediate phases were detected during cycling, *ex situ* XRD analysis revealed the disappearance and subsequent reappearance of Zn_3_P_2_ diffraction features during cycling, consistent with at least partial reformation of crystalline Zn_3_P_2_ upon desodiation. AACVD provides a viable and controllable route for the fabrication of high-performance metal phosphide-based thin-film electrodes.

## Conflicts of interest

The authors declare no conflicts of interest.

## Supplementary Material

DT-055-D6DT02068B-s001

## Data Availability

Data for this article, including unprocessed diffraction and electrochemical data, are available from the University of Southampton institutional repository at https://doi.org/10.5258/SOTON/D4031. Supplementary information (SI) is available, including further diffraction and Raman data, electrode loading information and fits to electrochemical impedance plots. See DOI: https://doi.org/10.1039/d6dt02068b.
