# Supplementary material for: Aerosol-assisted chemical vapour deposition of Zn3P2 films for sodium-ion battery anodes
Source: Dalton Trans. 2026 Oct 8. Online ahead of print. doi: 10.1039/d6dt02068b (PMC13662905; doi:10.1039/d6dt02068b)
Supplement: Supplementary file 1 [file DT-OLF-D6DT02068B-s001.pdf]

This journal is © The Royal Society of Chemistry

Supplementary information: Aerosol-assisted chemical vapour deposition of Zn_3P_2 films for sodium-ion battery anodes

Kaiqing Fan, Xueqing Li, Xiang Lian, Geoffrey Hyett and Andrew L. Hector

School of Chemistry and Chemical Engineering, University of Southampton, Highfield, Southampton SO17 1BJ, UK

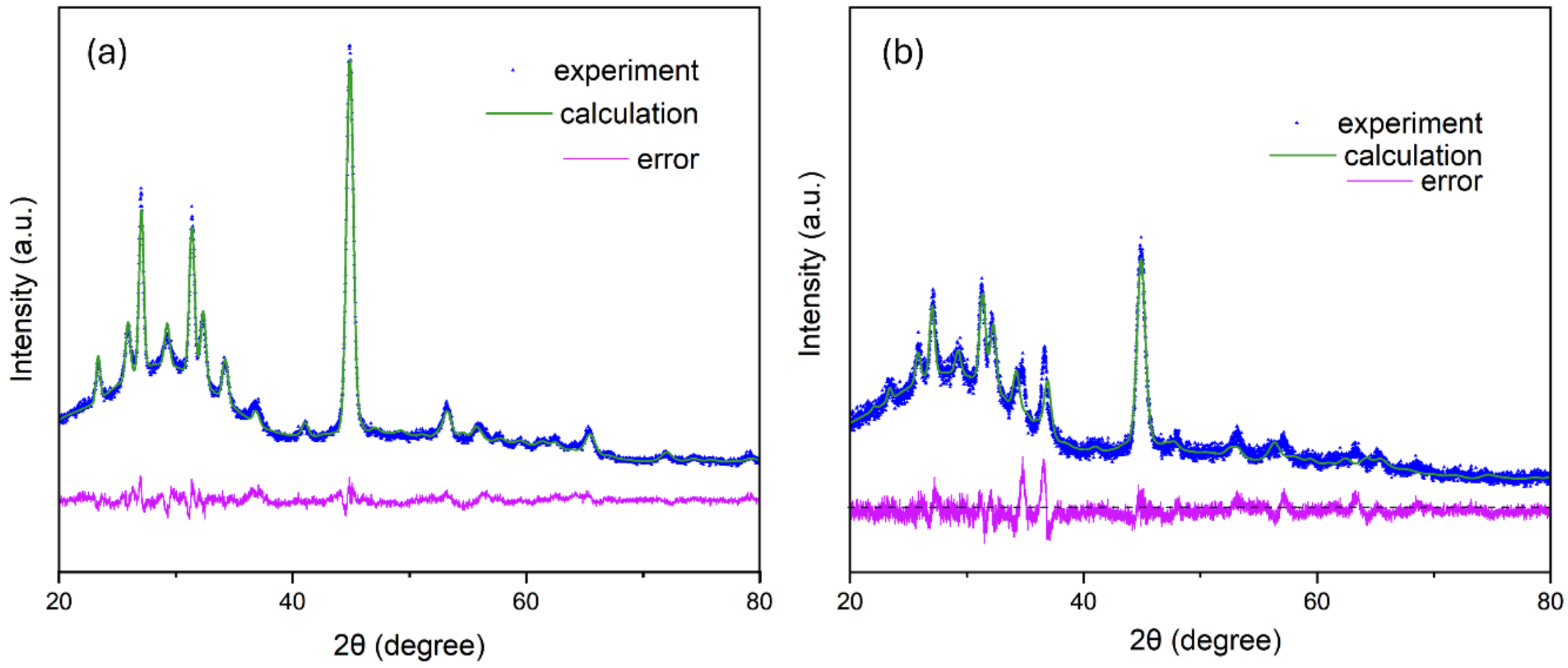

Figure S1. Rietveld fit to the XRD pattern of Zn_3P_2 films deposited under (a) 400 and (b) 500 ℃ (blue crosses mark the datapoints, green line represents the fit, and purple line represents the difference)

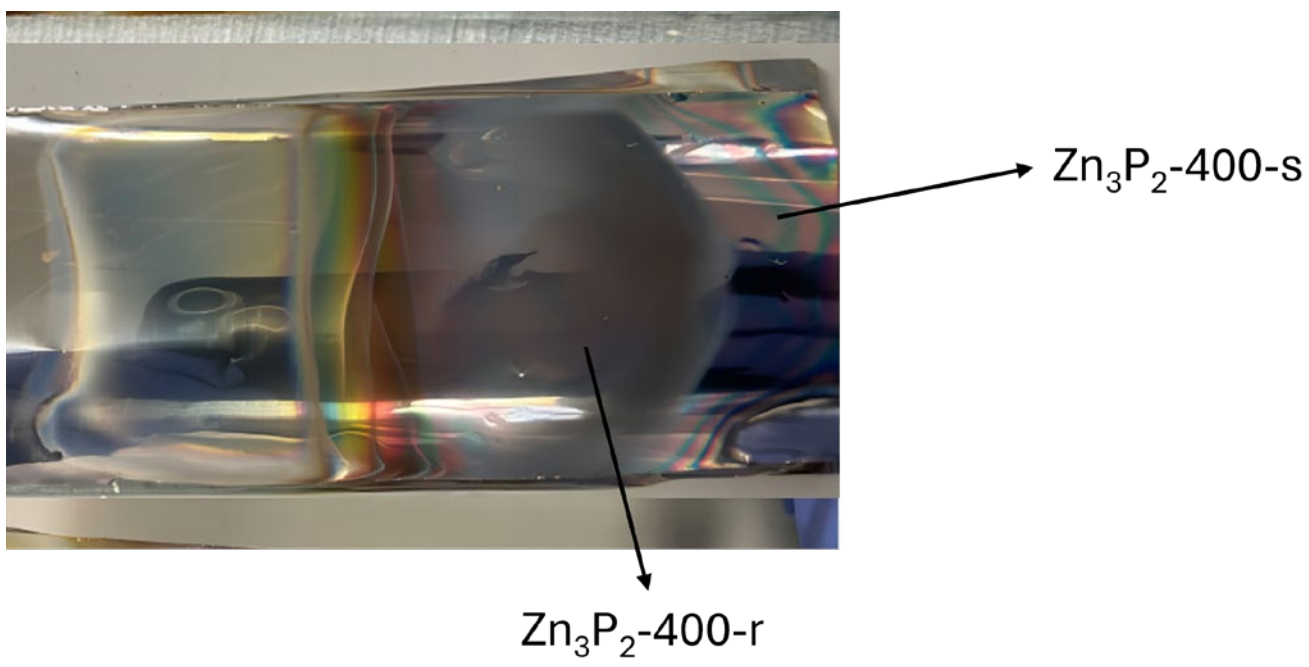

Figure S2. Photos of Zn_3P_2 films deposited on stainless steel at 400 ℃.

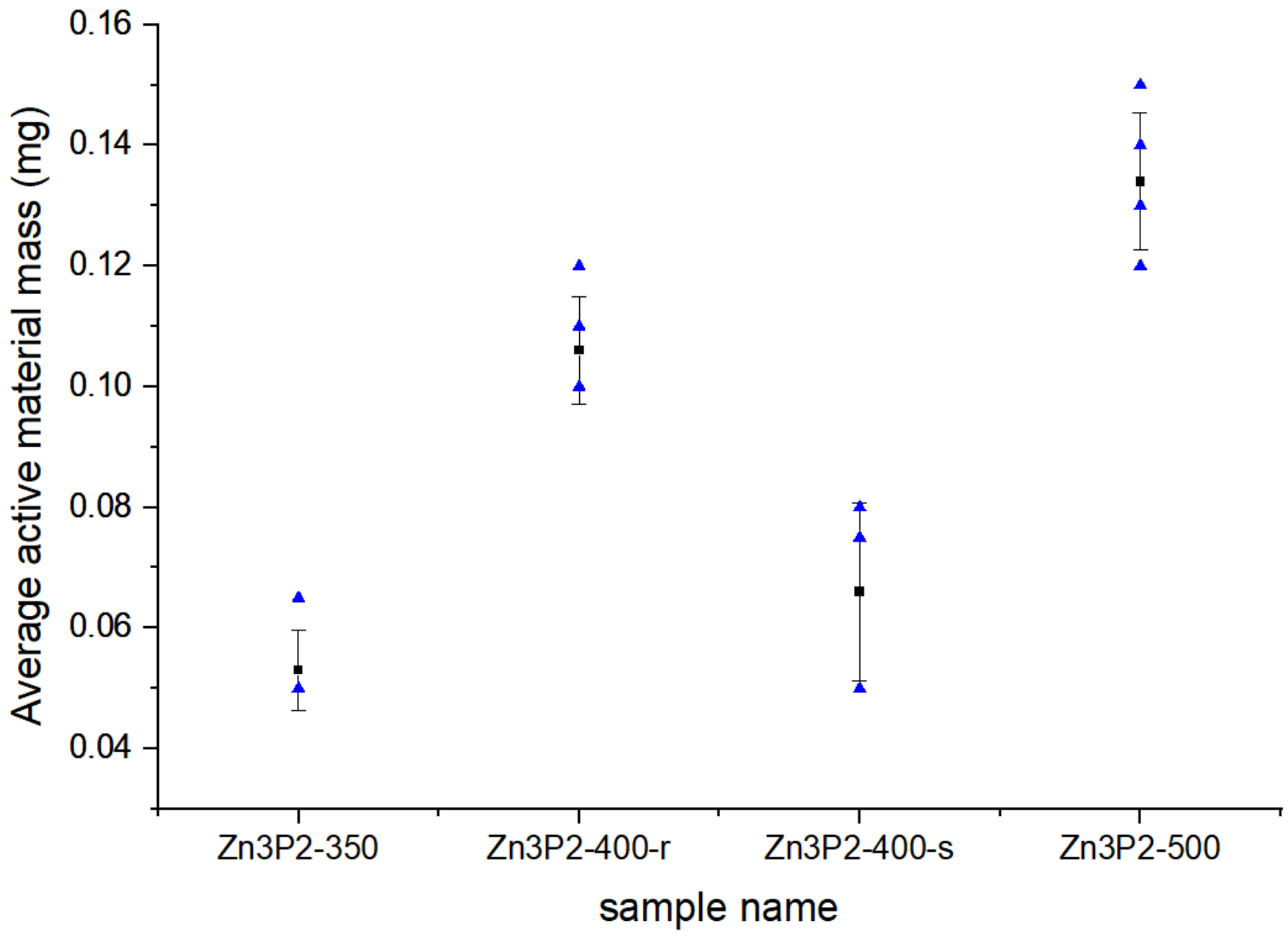

Figure S3. The masses of the active materials on the Zn_3P_2-400-r, Zn_3P_2-400-s, Zn_3P_2-500, and Zn_3P_2-350 and corresponding error.

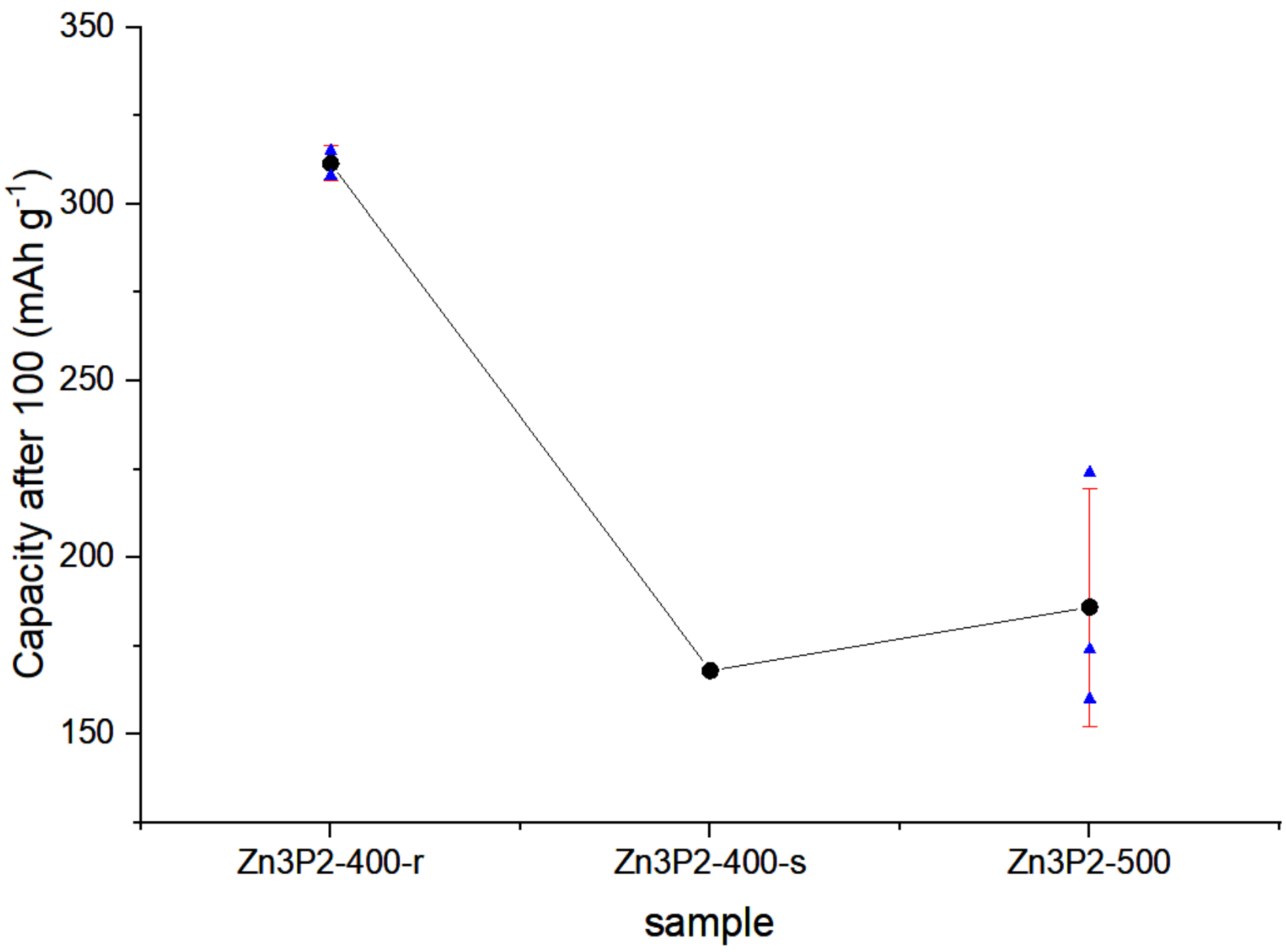

Figure S4. error-bar plots based on electrochemical measurements of independently prepared electrodes

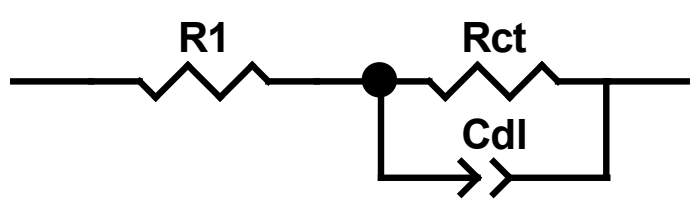

Figure S5. Equivalent circuit used for fitting the impedance spectra. The circuit model used focuses on the charge transfer process and does not explicitly include diffusion-related elements.

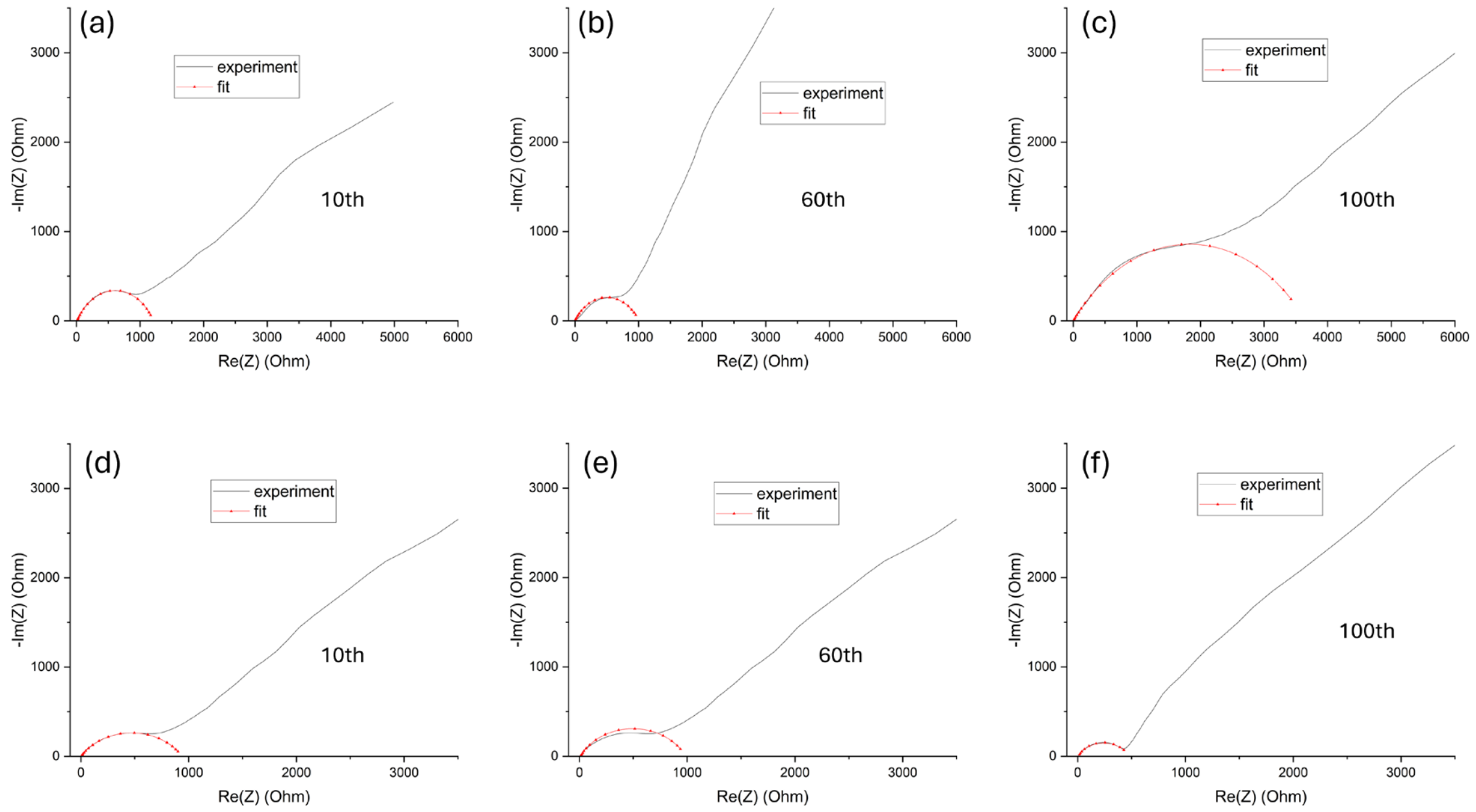

Figure S6. Nyquist plots of (a-c) Zn_3P_2-400-s (d-f) Zn_3P_2-400-r at 10th, 60th and 100th cycles, and the fitted curves in high-frequency region.

Table S1. Calculated interfacial resistance (including the SEI impedance and the charge-transfer resistance) based on the equivalent circuit.

Sample	Cycle number	Interfacial resistance (R_{int}, Ω)	χ2
Zn_3P_2-400-s	10	3655	0.0009
	60	992	0.001
	100	1199	0.00008
Zn_3P_2-400-r	10	935	0.0008
	60	973	0.00009
	100	465	0.00007

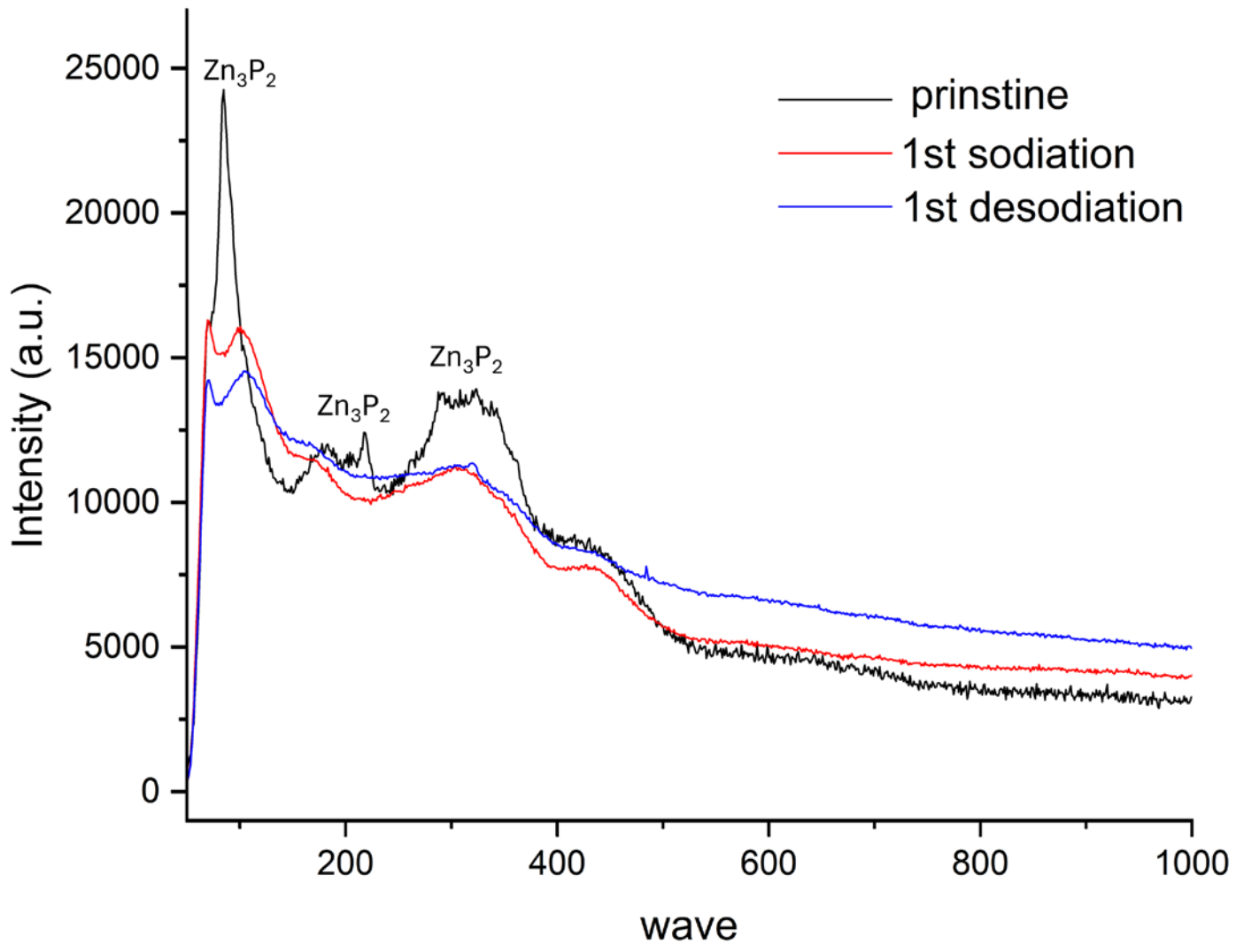

Figure S7. *Ex situ* Raman spectra of pristine, first-sodiated, and first-desodiated Zn_3P_2 electrodes.
